# Transition to Oral Antibiotic Therapy for Hospitalized Adults With Gram-Negative Bloodstream Infections

**DOI:** 10.1001/jamanetworkopen.2023.49864

**Published:** 2024-01-02

**Authors:** Drew W. Engers, Pranita D. Tamma, Suiyini Fiawoo, Karen Fong, Ripal Jariwala, Timothy C. Jenkins, Ronald E. Kendall, Jae Hyoung Lee, Erin K. McCreary, Payal K. Patel, Katherine C. Shihadeh, Judianne Slish, Trevor C. Van Schooneveld, Anurag N. Malani

**Affiliations:** 1 Department of Internal Medicine, Infectious Diseases, Trinity Health, Ann Arbor, Michigan; 2 Department of Pediatrics, The Johns Hopkins University School of Medicine, Baltimore, Maryland; 3 Department of Pharmacy, University of Utah Health, Salt Lake City; 4 Department of Pharmaceutical Services, University of California, San Francisco; 5 Department of Medicine, Division of Infectious Diseases, Denver Health, Denver, Colorado; 6 Department of Pharmacy, Veterans Affairs Ann Arbor Healthcare System, Ann Arbor, Michigan; 7 Department of Pediatrics, The Johns Hopkins University School of Medicine, Baltimore, Maryland; 8 Department of Medicine, Division of Infectious Diseases, University of Pittsburgh, Pittsburgh, Pennsylvania; 9 Department of Infectious Diseases, Intermountain Health, Salt Lake City, Utah; 10 Department of Pharmacy, Denver Health, Denver, Colorado; 11 Department of Pharmacy, University of Rochester Medicine–Highland Hospital, Rochester, New York; 12 Division of Infectious Diseases, Department of Internal Medicine, University of Nebraska Medical Center, Omaha

## Abstract

**Importance:**

Management of gram-negative bloodstream infections (GN-BSIs) with oral antibiotics is highly variable.

**Objective:**

To examine the transition from intravenous (IV) to oral antibiotics, including selection, timing, and associated clinical and microbial characteristics, among hospitalized patients with GN-BSIs.

**Design, Setting, and Participants:**

A retrospective cohort study was conducted of 4581 hospitalized adults with GN-BSIs at 24 US hospitals between January 1 and December 31, 2019. Patients were excluded if they died within 72 hours. Patients were excluded from the oral therapy group if transition occurred after day 7. Statistical analysis was conducted from July 2022 to October 2023.

**Exposures:**

Administration of antibiotics for GN-BSIs.

**Main Outcomes and Measures:**

Baseline characteristics and clinical parameters reflecting severity of illness were evaluated in groups receiving oral and IV therapy. The prevalence of transition from IV to oral antibiotics by day 7, median day of transition, sources of infection, and oral antibiotic selection were assessed.

**Results:**

Of a total of 4581 episodes with GN-BSIs (median age, 67 years [IQR, 55-77 years]; 2389 men [52.2%]), 1969 patients (43.0%) receiving IV antibiotics were transitioned to oral antibiotics by day 7. Patients maintained on IV therapy were more likely than those transitioned to oral therapy to be immunosuppressed (833 of 2612 [31.9%] vs 485 of 1969 [24.6%]; *P* < .001), require intensive care unit admission (1033 of 2612 [39.5%] vs 334 of 1969 [17.0%]; *P* < .001), have fever or hypotension as of day 5 (423 of 2612 [16.2%] vs 49 of 1969 [2.5%]; *P* < .001), require kidney replacement therapy (280 of 2612 [10.7%] vs 63 of 1969 [3.2%]; *P* < .001), and less likely to have source control within 7 days (1852 of 2612 [70.9%] vs 1577 of 1969 [80.1%]; *P* < .001). Transitioning patients from IV to oral therapy by day 7 was highly variable across hospitals, ranging from 25.8% (66 of 256) to 65.9% (27 of 41). A total of 4109 patients (89.7%) achieved clinical stability within 5 days. For the 3429 episodes (74.9%) with successful source control by day 7, the median day of source control was day 2 (IQR, 1-3 days) for the oral group and day 2 (IQR, 1-4 days) for the IV group (*P* < .001). Common infection sources among patients administered oral therapy were the urinary tract (1277 of 1969 [64.9%]), hepatobiliary (239 of 1969 [12.1%]), and intra-abdominal (194 of 1969 [9.9%]). The median day of oral transition was 5 (IQR, 4-6 days). Total duration of antibiotic treatment was significantly shorter among the oral group than the IV group (median, 11 days [IQR, 9-14 days] vs median, 13 days [IQR, 8-16 days]; *P* < .001]. Fluoroquinolones (62.2% [1224 of 1969]), followed by β-lactams (28.3% [558 of 1969]) and trimethoprim-sulfamethoxazole (11.5% [227 of 1969]), were the most commonly prescribed oral antibiotics.

**Conclusions and Relevance:**

In this cohort study of 4581 episodes of GN-BSIs, transition to oral antibiotic therapy by day 7 occurred in fewer than half of episodes, principally with fluoroquinolones, although this practice varied significantly between hospitals. There may have been additional opportunities for earlier and more frequent oral antibiotic transitions because most patients demonstrated clinical stability by day 5.

## Key Points

**Question:**

What is the timeline of transition from intravenous (IV) to oral antibiotics among patients with gram-negative bloodstream infections (GN-BSIs)?

**Findings:**

In this cohort study of 4581 GN-BSI episodes at 24 US hospitals, 43.0% of patients were transitioned to oral antibiotics by day 7; day 5 was the median day of oral transition, most commonly with fluoroquinolones (62.2%). Patients maintained on IV therapy had more severe illness and more comorbidities; however, 89.7% of patients were clinically stable within 5 days and the median day of source control was day 2 for all patients with successful source control by day 7.

**Meaning:**

This study suggests that, for patients with GN-BSIs, fewer than half of patients were transitioned from IV to oral therapy; opportunities exist for earlier and more frequent transitions.

## Teaser

This cohort study examines the transition from intravenous to oral antibiotics, including selection, timing, and associated clinical and microbial characteristics, among hospitalized patients with gram-negative bloodstream infections.

## Introduction

There is significant variability in the current management of gram-negative bloodstream infections (GN-BSIs).^1,2^ Given that GN-BSIs are common and contribute significantly to morbidity and mortality among hospitalized patients, addressing best practices for these patients is paramount.^3,4^ In addition, the recognition of emerging drug-resistant organisms and adverse effects of antibiotic use has amplified the importance of identifying appropriate therapeutic agents, duration of antibiotics, and routes of therapy.^5,6,7^

Several studies have examined commonly prescribed and effective durations of antibiotic therapy^8,9,10,11^; however, an understanding of clinician practices of transitioning from intravenous (IV) to oral antibiotic therapy for patients with GN-BSIs remains incomplete. Furthermore, while several surveys have queried clinicians’ practices regarding conversion from IV to oral therapy using hypothetical situations, the translation of survey responses to actual practice is unclear.^1,2^ There is likely significant variation in treatment practices across hospitals, influenced by factors such as availability of infectious diseases and antibiotic stewardship expertise and underlying patient characteristics, including severity of illness, immunosuppression, source of infection, source control, susceptible oral options, and antibiotic allergies. Prolonged treatment with IV antibiotics in the hospital or in the outpatient setting poses risks, such as phlebitis, venous thromboembolism, catheter occlusions, and catheter-related bloodstream infections, in addition to significant patient inconvenience and nursing workload.^7^ This study addresses practices with transition from IV to oral antibiotic therapy for the treatment of GN-BSI.

## Methods

### Study Objectives and Design

The primary objective of this cohort study was to characterize the current practice of timing and frequency of transitioning from IV to oral therapy by day 7 among adult patients hospitalized with GN-BSIs. The secondary objective was to compare demographic characteristics, clinical parameters, host factors, sources of infection, and antibiotic selection between the oral transition and IV therapy groups. The study was approved by the institutional review boards of the participating institutions with data use agreements in place with Johns Hopkins University. Patient consent waivers were granted as the study was retrospective and did not involve direct patient interaction. The Strengthening the Reporting of Observational Studies in Epidemiology (STROBE) reporting guideline was followed.^12^

Patients age 18 years or older hospitalized with GN-BSI between January 1 and December 31, 2019, at any of 24 US hospitals were included. Hospitals included a geographically diverse mix of academic (n = 16), community (n = 4), and Veterans Affairs medical centers (n = 4). The specific gram-negative organisms included are listed in Table 1. Subsequent GN-BSIs from the same patient were included if they occurred more than 90 days from the index infection. Patients who died or entered hospice within 72 hours of GN-BSI onset may not have had the opportunity to transition to oral therapy and were excluded.

**Table 1.** Baseline Demographic and Clinical Characteristics of Patients With Gram-Negative Bloodstream Infection Transitioned to Oral Antibiotic Therapy vs Continued Intravenous Antibiotics

Characteristic	Oral therapy, No. (%) (n = 1969)^a^	Intravenous therapy, No. (%) (n = 2612)	*P* value
Age, median (IQR), y	67 (54-77)	67 (55-76)	.26
Male	926 (47.0)	1463 (56.0)	<.001
Race and ethnicity			
Asian	91 (4.6)	119 (4.6)	.92
Black	404 (20.5)	642 (24.6)	.001
Hispanic	263 (13.4)	311 (11.9)	.14
White	1107 (56.2)	1406 (53.8)	.11
Other or multiracial^b^	104 (5.3)	134 (5.1)	.82
Weight, median (IQR), kg	77.5 (64.8-92.7)	76.7 (63.5-92.5)	.15
BMI, median (IQR)	27.8 (23.8-32.5)	26.6 (22.7-31.7)	<.001
<18.5	73 (3.7)	160 (6.1)	<.001
18.5-24.9	586 (29.8)	855 (32.7)	.03
25-29.9	567 (28.8)	715 (27.4)	.29
>30	743 (37.8)	882 (33.8)	.01
Charlson Comorbidty Index, median (IQR)^c^	2 (0-3)	2 (1-4)	<.001
Preexisting medical conditions			
Myocardial infarction	163 (8.3)	303 (11.6)	<.001
Congestive heart failure	208 (10.6)	418 (16.0)	<.001
Peripheral vascular disease	159 (8.1)	267 (10.2)	.01
Cerebrovascular disease	198 (10.1)	362 (13.9)	<.001
Dementia	128 (6.5)	240 (9.2)	.001
Chronic obstructive pulmonary disease	164 (8.3)	275 (10.5)	.01
Liver disease, moderate or severe	91 (4.6)	208 (8.0)	<.001
Diabetes	645 (32.8)	910 (34.8)	.14
Kidney disease^d^	438 (22.2)	656 (25.1)	.02
Immunosuppressed	485 (24.6)	833 (31.9)	<.001
Autoimmune condition receiving immunosuppressive therapy	118 (6.0)	183 (7.0)	.17
Solid tumor receiving chemotherapy	228 (11.6)	305 (11.7)	.92
Leukemia or multiple myeloma receiving chemotherapy	29 (1.5)	147 (5.6)	<.001
Lymphoma receiving chemotherapy	24 (1.2)	76 (2.9)	<.001
AIDS (CD4 count, <200 cells/mL)	15 (0.8)	24 (0.9)	.57
Solid organ transplant within the previous 12 mo	119 (6.0)	173 (6.6)	.43
Bone marrow transplant within the previous 12 mo	15 (0.8)	84 (3.2)	<.001
Graft-vs-host disease	1 (0.1)	25 (1.0)	<.001
Neutropenia (absolute neutrophil count, <500/mL during days 1-7 of gram-negative bloodstream infection)	47 (2.4)	204 (7.8)	<.001
Pitt bacteremia score, median (IQR)^e^	1 (0-2)	2 (1-3)	<.001
Intensive care unit admission	334 (17.0)	1033 (39.5)	<.001
Hypotension, median (IQR), days	0 (0-1)	0 (0-2)	<.001
Duration of fever, median (IQR), days	1 (0-2)	1 (0-2)	.16
Duration of bacteremia, median (IQR), days	1 (1-1)	1 (1-1)	<.001
Fever or hypotension on day 5 (without clinical stability)	49 (2.5)	423 (16.2)	<.001
Source control achieved within 7 d^f^	1577 (80.1)	1852 (70.9)	<.001
Source control day if achieved by day 7, median (IQR)^f^	2 (1-3)	2 (1-4)	<.001
Required kidney replacement therapy	63 (3.2)	280 (10.7)	<.001
Organism isolated from bloodstream			
*Acinetobacter* species	23 (1.2)	48 (1.8)	.21
*Citrobacter* species	28 (1.4)	53 (2.0)	.12
*Enterobacter* species	80 (4.1)	167 (6.4)	<.001
* Escherichia coli*	1189 (60.4)	1173 (44.9)	<.001
* Klebsiella/Enterobacter aerogenes*	26 (1.3)	21 (0.8)	.09
*Klebsiella* species	371 (18.8)	545 (20.9)	.09
* Morganella morganii*	6 (0.3)	35 (1.3)	<.001
*Proteus* species	76 (3.9)	160 (6.1)	.001
* Providencia stuartii*	5 (0.3)	6 (0.2)	.87
*Pseudomonas* species	115 (5.8)	286 (10.9)	<.001
*Serratia* species	39 (2.0)	103 (3.9)	<.001
* Stenotrophomonas maltophilia*	11 (0.6)	21 (0.8)	.32
ESBL producers	51 (2.6)	277 (10.6)	<.001
Duration of antibiotic therapy, median (IQR), d	11 (9-14)	13 (8-16)	<.001
Day transitioned to oral therapy, median (IQR)	5 (4-6)	NA	
Length of stay, median (IQR), d	4 (3-6)	12 (7-24)	<.001
30-d readmission	319 (16.2)	549 (21.0)	<.001

Abbreviations: BMI, body mass index (calculated as weight in kilograms divided by height in meters squared); ESBL, extended-spectrum β-lactamase; NA, not applicable.

^a^ Patients included in the oral therapy group were required to transition from intravenous antibiotics within 7 days.

^b^ No specific races or ethnicities were included in “Other.”

^c^ The Charlson Comorbidity Index generates a score ranging from 0 to 29 based on the number and severity of comorbid conditions, with increasing scores indicative of higher risk of death.^13^

^d^ Kidney disease was defined as chronic kidney disease or requiring kidney replacement therapy.

^e^ The Pitt bacteremia score ranges from 0 to 14 points, with a score 4 or more commonly used as an indicator of critical illness and increased risk of death.^14^

^f^ Source control was defined as drainage of all infected collections and/or removal of all infected hardware.

Day 1 was defined as the date of collection of the first positive blood culture result. Patients given oral therapy by day 7 were included in the oral transition group. Patients maintained on IV therapy as of day 7 were included in the IV therapy group. From these 2 groups, baseline characteristics and clinical variables were assessed. The prevalence of oral transition was defined as GN-BSI episodes that had transition to oral therapy by day 7. This time limit for evaluating patients’ transition to oral therapy was chosen given evidence favoring the duration of only 7 days of antibiotic therapy for uncomplicated GN-BSIs.^8,9,10^ Additional antibiotic therapy after day 7, regardless of whether IV or oral, was deemed unlikely to be associated with an incremental clinical benefit, based on the results of multiple studies and expert opinion.^8,11,15,16^

### Data Collection

Electronic medical record reviews were conducted manually at all 24 sites and submitted into a secure REDCap (Research Electronic Data Capture) database. The following data were collected: demographic characteristics, including age, sex, and race and ethnicity as reported in the electronic health record (Alaska Native or American Indian, Asian, Black, Hispanic, Native Hawaiian or Pacific lslander, White, and other or multiracial [no specific races or ethnicities were included in “other”]); comorbidities (including myocardial infarction, congestive heart failure, peripheral vascular disease, cerebrovascular disease, dementia, chronic obstructive pulmonary disease, moderate or severe liver disease, diabetes, kidney disease [defined as chronic kidney disease and/or kidney replacement therapy], autoimmune conditions, malignant neoplasms and active chemotherapy, AIDS, solid organ transplant, bone marrow transplant, and neutropenia); Charlson Comorbidity Index^13^ (with a score ranging from 0 to 29 based on the number and severity of comorbid conditions, with increasing scores indicative of higher risk of death); clinical status (including Pitt bacteremia score^14^ [ranges, 0-14 points, with a score ≥4 commonly used as an indicator of critical illness and increased risk of death], hemodynamic instability, duration of fever, intensive care unit (ICU) admission on the day of or within 1 day of positive blood culture result); source of infection and source control status (defined as removal of all infected hardware and/or drainage of all infected collections); microbiology test results (bacterial genus and species, presence of antimicrobial resistance, and duration of bacteremia); antibiotic regimens; and patient outcomes. Race and ethnicity were included as collected from the electronic health record to address population diversity and generalizability. All data were stored in a confidential database through Johns Hopkins University with deidentified patient information.

### Statistical Analysis

Statistical analysis was conducted from July 2022 to October 2023 and was largely descriptive to understand the prevalence of transitioning to oral therapy for GN-BSIs, common regimens prescribed, and variables associated with an increased likelihood of oral transition. Continuous and categorical variables comparing the IV and oral transition groups were compared using the Mann-Whitney *U* test and the χ^2^ test, respectively. All *P* values were from 2-sided tests and results were deemed statistically significant at *P* < .05. Statistical analyses were conducted using Stata, version 17 software (StataCorp LLC).

## Results

Among 4581 episodes with GN-BSI across the 24 sites, the median (IQR) age of patients was 67 years (IQR, 55-77 years) (IV: median, 67 years [IQR, 54-77 years] and oral: median, 67 years [IQR, 55-76 years]; *P* = .26), there were 2389 men (52.2%) and 2192 women (47.8%), and the study cohort included 210 Asian individuals (4.6%), 1046 Black individuals (22.8%), 574 Hispanic individuals (12.5%), and 2513 White individuals (54.9%) (Table 1). There were 264 episodes of GN-BSI excluded because these patients had died or entered hospice within 72 hours of GN-BSI onset. The IV therapy cohort included more men (1463 of 2612 [56.0%] vs 926 of 1969 [47.0%]; *P* < .001) and a greater proportion of Black patients (642 of 2612 [24.6%] vs 404 of 1969 [20.5%]; *P* = .001) compared with the oral therapy cohort. The median Charlson Comorbidity Index was 2 (IQR, 0-3) in the oral group and 2 (IQR, 1-4) in the IV group (*P* < .001). A total of 1969 patients (43.0%) cases transitioned to oral antibiotics by day 7. The median day of oral transition was 5 (IQR, 4-6 days).

Several patient characteristics and clinical variables were assessed in the oral therapy cohort and those maintained on IV therapy (Table 1). The IV therapy group had a higher prevalence of numerous medical conditions, including myocardial infarction, congestive heart failure, peripheral vascular disease, cerebrovascular disease, chronic obstructive pulmonary disease, dementia, liver disease, and kidney disease. The IV group also had more patients with underlying immunosuppression compared with the oral group (833 of 2612 [31.9%] vs 485 of 1969 [24.6%]; *P* < . 001). Clinical characteristics were also associated with prolonged IV therapy, with ongoing hypotension or fevers more common by day 5 among the IV group compared with the oral therapy group (423 of 2612 [16.2%] vs 49 of 1969 [2.5%]; *P* < .001), although most did not have fever or hypotension as of day 5 (4109 [89.7%]). Source control was achieved within 7 days for 3429 episodes (74.9%) and was more frequent among the oral therapy group compared with the IV therapy group (1577 of 1969 [80.1%] vs 1852 of 2612 [70.9%]; *P* < .001). Of cases with successful source control by day 7, the median day was 2 (IQR, 1-3 days) for the oral group and 2 (IQR, 1-4 days) for the IV group. More than twice as many patients who were given and maintained on IV therapy were admitted to the ICU compared with those transitioned to oral therapy (1033 of 2612 [39.5%] vs 334 of 1969 [17.0%]; *P* < .001). The median Pitt bacteremia score was higher for the IV therapy group than the oral therapy group (2 [IQR, 1-3] vs 1 [IQR, 0-2]; *P* < .001). Kidney replacement therapy was more common among the IV therapy group compared with the oral transition group (280 of 2612 [10.7%] vs 63 of 1969 [3.2%]; *P* < .001).

**Figure 1.**
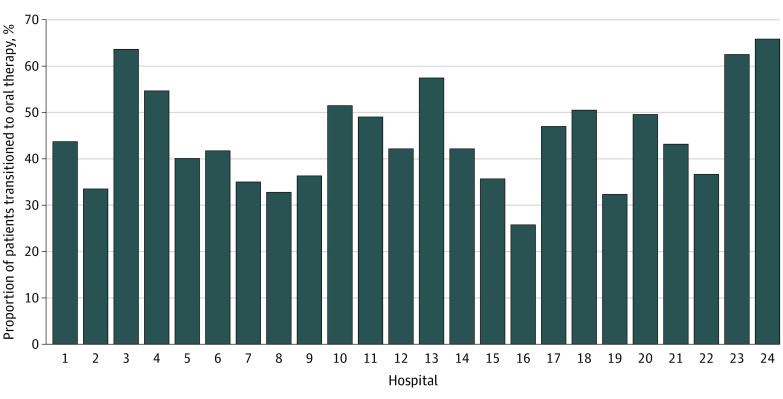
Percentage of Patients Transitioned to Oral Antibiotic Therapy for Gram-Negative Bloodstream Infections by Day 7

**Figure 2.**
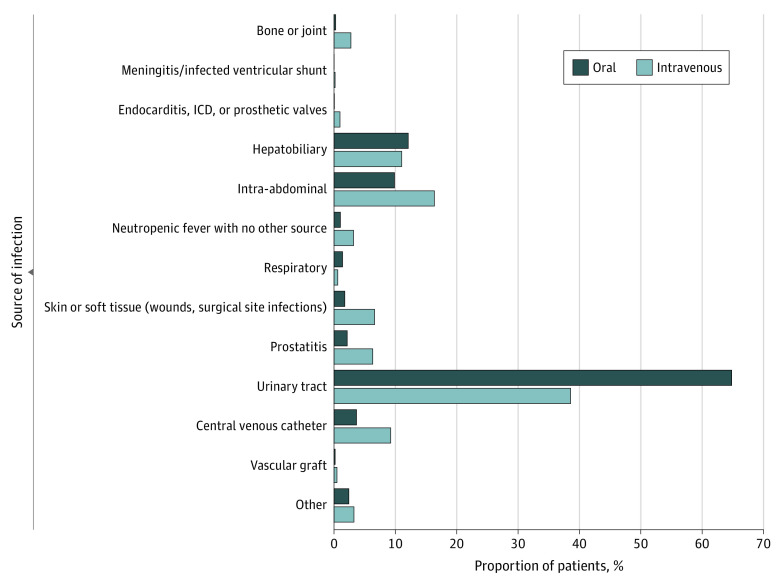
Source of Infection Among Patients Transitioned to Oral Antibiotic Therapy vs Intravenous Therapy ICD indicates implantable cardiac device.

The proportion of patients transitioned from IV to oral therapy by day 7 for GN-BSI was highly variable among the 24 hospitals. The lowest rate of oral transition was 25.8% (66 of 256), the highest was 65.9% (27 of 41), and most were between 30% and 50% (Figure 1; eTable 1 in Supplement 1). The most common source of infection among patients administered oral therapy was the urinary tract, with *Escherichia coli* as the most common organism (60.4% [1189 of 1969]). Urinary tract infections (UTIs) comprised 64.9% (1277 of 1969) of all infections among the oral therapy group, followed by hepatobiliary (12.1% [239 of 1969]) and intra-abdominal (9.9% [194 of 1969]) infections (Figure 2; eTable 2 in Supplement 1). Among patients maintained on IV therapy, UTIs were similarly the most common infection (38.6% [1008 of 2612]), followed by intra-abdominal (16.4% [429 of 2612]), hepatobiliary (11.1% [289 of 2612]), and central venous catheter (9.3% [242 of 2612]) infections. Of patients transitioned to oral therapy by day 7, commonly isolated organisms were *E coli* (60.4% [1189 of 1969]), *Klebsiella* species (18.8% [371 of 1969]), *Pseudomonas* species (5.8% [115 of 1969]), *Enterobacter* species (4.1% [80 of 1969]), *Proteus* species (3.9% [76 of 1969]), and *Serratia* species (2.0% [39 of 1969]) (Table 1). There were more extended-spectrum β-lactamase–producing bacterial infections in the IV therapy group compared with the oral therapy group (10.6% [277 of 2612] vs 2.6% [51 of 1969]; *P* < .001). Several other organisms were more prevalent in the IV group than the oral therapy group, including *Enterobacter* species (6.4% [167 of 2612] vs 4.1% [80 of 1969]; *P* < .001), *Morganella morganii* (1.3% [35 of 2612] vs 0.3% [6 of 1969]; *P* < .001), *Proteus* species (6.1% [160 of 2612] vs 3.9% [76 of 1969]; *P* = .001), *Pseudomonas* species (10.9% [286 of 2612] vs 5.8% [115 of 1969]; *P* < .001), and *Serratia* species (3.9% [103 of 2612] vs 2.0% [39 of 1969]; *P* < .001). Total median duration of antibiotic therapy was significantly shorter for the oral transition group than the IV group (11 days [IQR, 9-14 days] vs 13 days [IQR, 8-16]; *P* < .001).

Of the available options for oral transition therapy, fluoroquinolones (62.2% [1224 of 1969]), β-lactams (28.3% [558 of 1969]), and trimethoprim-sulfamethoxazole (TMP-SMX) (11.5% [227 of 1969]) were the most commonly prescribed. Cephalosporins (20.7% [408 of 1969]) were prescribed more frequently than aminopenicillins (7.6% [150 of 1969]) (Table 2).

**Table 2.** Oral Antibiotics Selected for Transition Therapy

Antibiotic	Patients receiving treatment, No. (%) (n = 1969)^a^
Fluoroquinolones	1224 (62.2)
Ciprofloxacin	973 (49.4)
Levofloxacin	246 (12.5)
Moxifloxacin	5 (0.3)
Trimethoprim-sulfamethoxazole	227 (11.5)
β-Lactams	558 (28.3)
Amoxicillin, amoxicillin-clavulanate	150 (7.6)
Cephalosporin (cefadroxil, cefdinir, cefpodoxime, cefuroxime, cephalexin)	408 (20.7)
Other: tetracyclines (doxycycline, minocycline), nitrofurantoin	17 (0.9)

^a^ A total of 47 patients received more than 1 oral antibiotic.

## Discussion

This study examined the current practices of oral transition therapy for patients with GN-BSIs in 24 US hospitals. Fewer than half the patients in this cohort (43.0%) were provided oral transition therapy within 7 days and the median day of oral transition was 5 (IQR, 4-6 days). There are many facets of care and antimicrobial stewardship to consider as possible reasons that oral transition was deferred. Achieving clinical improvement and source control are routinely assessed before considering this transition. In this study, clinical stability with resolution of fever or hypotension was common among both the oral transition and IV groups by day 5. Approximately 90% of patients had achieved clinical stability by day 5 and approximately 75% had source control within the first week (median, 2 days), yet fewer than half were provided oral therapy by day 7. These results suggest there may be significant opportunities for early and more frequent oral antibiotic transitions. Ongoing bacteremia did not appear to be a limiting factor as both groups had a median of 1 day of bacteremia. Host factors appeared to be associated with the decision to transition from IV to oral therapy, with numerous comorbidities associated with prolonged IV therapy. In particular, underlying immunosuppression was present in 31.9% of the IV group compared with 24.6% of the oral transition group. The discrepancy between patients who should be able to receive oral therapy and those who actually received it becomes even more notable when considering the variability in oral transition practices between hospitals (Figure 1). Survey studies have found considerable practice variation in the management of GN-BSIs,^1,2^ especially with transitions from IV to oral therapy, and our study confirms these findings with real-world data.

Most GN-BSI episodes were from urinary sources with Enterobacterales, an area of practice with robust literature supporting the safety and efficacy of oral transition therapy. Data from the UTI literature, including randomized clinical trials, demonstrate the utility of oral antibiotics and treatment duration of 7 days.^17,18^ A retrospective analysis examining bacteremic Enterobacteriaceae UTIs did not demonstrate any differences in treatment failure when patients were transitioned to oral therapy.^19^ Another retrospective Enterobacterales bacteremia study showed that clinical outcomes with oral transition compared with continued IV therapy did not demonstrate any differences in 30-day all-cause mortality or recurrence of bacteremia and was associated with a decreased length of stay.^20^ With the knowledge that safe and effective clinical outcomes are possible in oral transition therapy, the current study suggests that many of the patients in our cohort may have had options for an earlier transition to oral therapy with a higher frequency than what was observed. Antimicrobial stewardship teams can consider developing targeted education on when transition to oral antibiotics can be considered for GN-BSIs that address patient identification, timing of transition, agent selection, and duration of therapy.

An ongoing controversy is the optimal oral agent. A small prospective study evaluated the transition from IV to oral ciprofloxacin for GN-BSIs and found comparable clinical resolution, few toxic effects, significantly shorter length of stay, and decreased costs.^21^ In this study, fluoroquinolones were the preferred agent. Although oral transition to highly orally bioavailable agents such as fluoroquinolones or TMP-SMX was observed, β-lactam agents were not infrequently chosen by practitioners. There is ongoing debate about β-lactam bioavailability, absorption, and pharmacokinetics, yet multiple studies have addressed clinical outcomes. A meta-analysis of patients with Enterobacterales bacteremia who were transitioned to oral β-lactams vs fluoroquinolones or TMP-SMX did not find any significant differences in all-cause mortality between the 2 groups.^22^ Another study of bacteremia with a urinary source noted that the risk of recurrent bacteremia treated with oral β-lactams was not higher than the risk for those treated with TMP-SMX or fluoroquinolones and there were no mortality differences.^23^ Future analysis by this collaborative will attempt to address areas of treatment with oral β-lactams that likely affect success rates, such as appropriate dosing regimens and the time of transition.

### Limitations

Our study has several limitations. There was potential for selection bias in this collaborative given that participating hospitals had engaged and experienced antimicrobial stewardship programs. However, the data were collected from a diverse array of institutions throughout the US, and the large cohort size likely has generalized and validated findings. There were major differences between patients who transitioned to oral therapy and those maintained on IV treatment. The IV group included patients with more underlying comorbidities and a higher severity of illness (Pitt bacteremia score, ongoing fever or hypotension by day 5, ICU admission, immunosuppression, lack of source control, kidney replacement therapy), which were presumably associated with the oral transition decision, but the individual contribution of these various factors was not assessed. Although we explored an exhaustive list of variables and comorbidities associated with clinician decisions to transition patients from IV to oral therapy, other unrecognized factors may have been associated with these decisions, such as drug allergies, drug interactions, and the ability to absorb oral therapy, which were not captured. The practice of continuing IV therapy while the patient is in the hospital and transitioning to oral antibiotics on discharge is common; thus, other reasons for which patients stayed in the hospital may have been associated with the timing of transition to oral therapy. This analysis did not assess if there was a lack of active oral agents in each episode that precluded oral transition, which may explain why those with *Pseudomonas* or extended-spectrum β-lactamase pathogens that have limited oral treatment options typically continued to receive IV therapy. Finally, most GN-BSI episodes in this cohort were secondary to the urinary tract, which may limit generalizability to all cases of GN-BSI. Future prospective research is also needed to provide additional data for developing evidence-based guidelines on the optimal approach to transition to oral antibiotic therapy in GN-BSIs.

## Conclusions

This study delivers insight into the importance of further antibiotic stewardship interventions in the management of adult hospitalized patients with GN-BSIs. Oral transition therapy occurred for fewer than half of eligible patients, principally with fluoroquinolones, although this varied significantly based on hospital site. There appear to be opportunities for earlier and more frequent oral antibiotic transitions because most patients demonstrated clinical stability by day 5.

## Notes

Supplement 1. **eTable 1.** Patients With Intravenous Therapy vs Oral Therapy Transition by Day 7 for Gram-Negative Bloodstream Infections in Cohort Hospitals **eTable 2.** Source of Infection in Patients Transitioned to Oral Antibiotic Therapy vs Intravenous Therapy

Supplement 2.
Data Sharing Statement
