# Supplementary material for: Transition to Oral Antibiotic Therapy for Hospitalized Adults With Gram-Negative Bloodstream Infections
Source: JAMA Netw Open. 2024 Jan 2;7(1):e2349864. doi: 10.1001/jamanetworkopen.2023.49864 (PMC10762571; doi:10.1001/jamanetworkopen.2023.49864)
Supplement: Supplement 1. — eTable 1. Patients With Intravenous Therapy vs Oral Therapy Transition by Day 7 for Gram-Negative Bloodstream Infections in Cohort Hospitals eTable 2. Source of Infection in Patients Transitioned to Oral Antibiotic Therapy vs Intravenous Therapy [file jamanetwopen-e2349864-s001.pdf]

Supplementary Online Content

Engers DW, Tamma PD, Fiawoo S, et al. Transition to oral antibiotic therapy for hospitalized adults with gram-negative bloodstream infections. *JAMA Netw Open.* 2024;7(1):e2349864. doi:10.1001/jamanetworkopen.2023.49864

eTable 1. Patients With Intravenous Therapy vs Oral Therapy Transition by Day 7 for Gram-Negative Bloodstream Infections in Cohort Hospitals

eTable 2. Source of Infection in Patients Transitioned to Oral Antibiotic Therapy vs Intravenous Therapy

This supplementary material has been provided by the authors to give readers additional information about their work.

© 2024 Engers DW et al. *JAMA Network Open.*

eTable 1. Patients With Intravenous Therapy vs Oral Therapy Transition by Day 7 for Gram-Negative Bloodstream Infections in Cohort Hospitals

**Hospital**	**IV therapy**	**Oral therapy transition**	**Total patients**	**Percent transitioned to oral therapy**
1	89	69	158	43.7%
2	113	57	170	33.5%
3	71	124	195	63.6%
4	103	124	227	54.6%
5	136	91	227	40.1%
6	211	151	362	41.7%
7	275	148	423	35.0%
8	80	39	119	32.8%
9	177	101	278	36.3%
10	65	69	134	51.5%
11	156	150	306	49.0%
12	221	161	382	42.2%
13	89	120	209	57.4%
14	81	59	140	42.1%
15	177	98	275	35.6%
16	190	66	256	25.8%
17	26	23	49	46.9%
18	102	104	206	50.5%
19	67	32	99	32.3%
20	115	113	228	49.6%
21	29	22	51	43.1%
22	19	11	30	36.7%
23	6	10	16	62.5%
24	14	27	41	65.9%
**Total**	2612	1969	4581	43.0%

© 2024 Engers DW et al. *JAMA Network Open.*

eTable 2. Source of Infection in Patients Transitioned to Oral Antibiotic Therapy vs Intravenous Therapy

Source of Infection	Oral Therapy Transition, No. (%), n = 1969	IV Therapy, No. (%), n = 2612
Bone or joint	6 (0.3)	73 (2.8)
Meningitis/Infected Ventricular shunt	0 (0)	5 (0.2)
Endocarditis/ICD/Prosthetic valves	1 (0.05)	26 (1)
Hepatobiliary	239 (12.1)	289 (11.1)
Intra-abdominal	194 (9.9)	429 (16.4)
Neutropenic fever with no other identified source	21 (1.1)	84 (3.2)
Respiratory	28 (1.4)	17 (0.7)
Skin or soft tissue (wounds, surgical site infections)	35 (1.8)	174 (6.7)
Prostatitis	43 (2.2)	166 (6.4)
Urinary tract	1277 (64.9)	1008 (38.6)
Central venous catheter	73 (3.7)	242 (9.3)
Vascular graft	4 (0.2)	13 (0.5)
Other	48 (2.4)	86 (3.3)

© 2024 Engers DW et al. *JAMA Network Open.*
