# Supplementary material for: Transition to Oral Antibiotic Therapy for Hospitalized Adults With Gram-Negative Bloodstream Infections
Source: JAMA Netw Open. 2024 Jan 2;7(1):e2349864. doi: 10.1001/jamanetworkopen.2023.49864 (PMC10762571; doi:10.1001/jamanetworkopen.2023.49864)
Supplement: Supplement 2. — Data Sharing Statement [file jamanetwopen-e2349864-s002.pdf]

Data Sharing Statement

Engers. Transition to Oral Antibiotic Therapy for Hospitalized Adults With Gram-Negative Bloodstream Infections. *JAMA Netw Open*. Published January 02, 2024. doi:10.1001/jamanetworkopen.2023.49864

Data
Data available: No
